# Comparison and predictors of implantable cardioverter-defibrillator therapy for primary and secondary prevention

**DOI:** 10.1007/s12471-023-01785-0

**Published:** 2023-06-16

**Authors:** Reinder Evertz, Tessa van der Heijden, Rypko Beukema, Sjoerd Westra, Esther Meindersma, Caroline van Deursen, Kevin Vernooy

**Affiliations:** 1 Department of Cardiology, Radboud University Medical Centre, Nijmegen, The Netherlands; 2 Department of Cardiology, Cardiovascular Research Institute Maastricht, Maastricht University Medical Centre, Maastricht, The Netherlands

**Keywords:** Implantable cardioverter-defibrillator therapy, Primary prevention, Secondary prevention, Mortality

## Abstract

**Background:**

Implantable cardioverter-defibrillators (ICDs) are effective in detecting and treating ventricular arrhythmias. Studies on ICD therapy for different indications (primary and secondary prevention) and possible predictors of ICD therapy are limited. In this study, the incidence and type of ICD therapy were related to the indication and the underlying cardiac pathology.

**Methods:**

A single-centre, retrospective and observational study was performed of 482 patients who underwent ICD implantation for primary (53.3%) or secondary prevention (46.7%) between 2015 and 2020 at the Radboud University Medical Centre.

**Results:**

During a median follow-up of 2.4 years (interquartile range 0.2–3.9), the occurrence of appropriate ICD therapy for primary versus secondary prevention was 9.7% and 27.6%, respectively (*p* < 0.001). Time to appropriate ICD therapy was significantly shorter in the secondary prevention group (*p* < 0.001). No difference in ICD therapy was seen for different underlying aetiologies. In the majority of cases (70%) ICD therapy was given for ventricular tachycardia (VT). The occurrence of adverse events (16.3% vs 17.3%, *p* = 0.772), hospitalisation for cardiovascular reasons (29.2% vs 35.1%, *p* = 0.559) and all-cause mortality (12.5% vs 11.6%, *p* = 0.763) were similar in both groups. Male gender (3.53, 95% confidence interval (CI) (1.003, 12.403), *p* = 0.049) and secondary prevention indication (4.90, 95% CI (1.495, 16.066), *p* = 0.009) were predictors of appropriate ICD therapy.

**Conclusion:**

The risk associated with appropriate ICD therapy is higher in secondary prevention patients, who have their first therapy within a shorter time frame after device implantation. Rates of complications, hospitalisation and all-cause mortality are comparable. Future treatment options should target the prevention of ICD therapy, mainly by preventing the recurrence of VT.

## What’s new?

- In this retrospective observational study the appropriate implantable cardioverter-defibrillator (ICD) therapy for both primary and secondary prevention has been analysed.
- Although the incidence of appropriate ICD therapy differed significantly between primary and secondary prevention, all-cause mortality did not.
- Knowledge of the ICD therapy has implications as regards the future prevention of ICD therapy and therefore improving the quality of life of these patients.

## Introduction

**Fig. 1:**
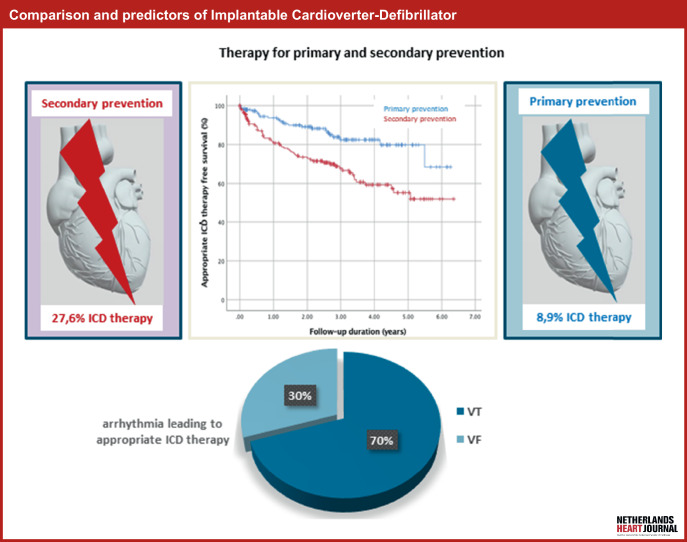
Infographic

Sudden cardiac death (SCD) is an important cause of mortality and occurs within an hour after symptoms have developed. In most cases, SCD is due to ventricular arrhythmias, most often associated with underlying ischaemic, non-ischaemic or electrical heart disease [1]. Implantable cardioverter-defibrillators (ICDs) are effective in detecting and terminating potentially life-threatening ventricular arrhythmias. As a result, ICDs prevent SCD. An ICD for primary prevention is indicated in patients who have not experienced ventricular arrhythmia but have cardiac pathology that places them at a higher risk of developing such arrhythmia. Secondary prevention entails ICD implantation in survivors of ventricular fibrillation (VF) or patients with sustained ventricular tachycardia (VT) in structural heart disease [2]. Although an ICD effectively prevents SCD, it does not prevent arrhythmic events. An ICD can treat ventricular arrhythmias with anti-tachycardia pacing (ATP), an ICD shock, or both. Administration of an ICD shock differs dramatically from ATP and has a different impact on the patient’s physical and psychological well-being. Data that compare the occurrence of (appropriate) ICD therapy for the indications are scarce. These studies demonstrate that appropriate ICD therapy (including ATP and ICD shock) occurs more often among patients with an ICD for secondary prevention [3–6]. Nevertheless, this effect disappeared when the incidence of appropriate therapy was determined for ICD shock only. Previous studies have also identified potential predictors of ICD therapy. However, the results were often heterogeneous [5]. Schaer et al. assessed possible predictors of ICD therapy in patients with an ICD for secondary prevention. ICD implantation for VT and age were predictors of overall ICD therapy [4]. This study aimed to assess ICD therapy in different patient groups, based on indication (primary vs secondary) or underlying heart disease (e.g. ischaemic or non-ischaemic heart disease). The type of ICD therapy could have therapeutic implications as regards improving the future care of these patients by preventing the occurrence of ventricular arrhythmias and thus reducing the need for ICD therapy (Fig. 1).

## Patients and methods

### Study population

A total of 502 patients who underwent ICD implantation for primary or secondary prevention between 2015 and 2020 at the Radboud University Medical Centre, Nijmegen (Radboudumc) were enrolled in this retrospective, observational study. These patients were registered with their medical record number. All necessary data were collected from their medical records and device interrogation. Primary prevention was defined as ICD implantation in patients with symptomatic heart failure and a left ventricular ejection fraction (LVEF) of ≤ 35% despite optimal medical therapy, and who were at least 40 days post-myocardial infarction or had non-ischaemic cardiomyopathy with an LVEF of ≤ 35%. Secondary prevention was defined as ICD implantation in patients who survived cardiac arrest due to VF or in patients with sustained VT in structural heart disease [2]. Patients who were not seen regularly or for whom follow-up data (ICD or medical) were not available were excluded from this study. Patients younger than 18 years at the time of implantation or who had stated that they did not want their data used for scientific research were also excluded.

### Methods

Data were obtained from the patients’ medical records and via device interrogation and were analysed retrospectively. Analysis of the device interrogation was performed in order to characterise the type of therapy (ATP, ICD shock, or both), whether it was for VT or VF, and whether it was appropriate or inappropriate. ICD therapy because of ventricular arrhythmia was defined as appropriate therapy. Therapy for arrhythmias other than ventricular arrhythmia or administered due to technical issues was defined as inappropriate. The relevant episode data, such as duration of the arrhythmia, time to diagnosis and the therapy given, were reviewed for each episode. The revision and assessment of the stored ICD therapy was done by a qualified electrophysiologist. The study was approved by the local ethics committee (file number 2021–8215).

### Study outcomes

The primary outcome was the incidence, time to first and difference in the type of ICD therapy during follow-up for different indications (primary vs secondary prevention) and underlying heart disease (ischaemic vs non-ischaemic). Secondary outcomes were the incidence of inappropriate ICD therapy, complications (access-, lead- or device-related, or infection), hospitalisation for cardiovascular disease and all-cause mortality (cardiac and non-cardiac).

### Statistical analysis

In this study, categorical or continuous variables were used. Categorical data were presented as absolute numbers and percentages. Continuous variables were expressed as mean ± SD or median (interquartile range (IQR) Q1–Q3) for skewed distributions. Differences at baseline and in ICD therapy were compared for primary versus secondary prevention and ischaemic versus non-ischaemic heart disease. These data were analysed using a Student’s *t*-test or Mann-Whitney test for the numerical variables and a chi-square test or Fisher’s exact test for the categorical variables. The secondary outcomes were also analysed with a chi-square or Fisher’s exact test. In the results, ‘appropriate first ICD therapy’ implied that the first ICD therapy administered was appropriate. The time to appropriate ICD therapy and all-cause mortality since implantation were analysed with the Kaplan-Meier method and compared using the log rank test. Uni-and multivariable binary logistic regression analyses were performed to determine possible predictors of appropriate ICD therapy during follow-up. All baseline characteristics and the indication (primary vs secondary) were analysed individually in a univariable binary logistic regression. Significant univariable predictors were included in the multivariable logistic regression using the enter method and the forward LR method. The statistical analyses were performed using SPSS Statistics, Version 25 (IBM Corp., Armonk, NY, USA). All the *p*-values were two-sided, and a *p*-value < 0.05 was considered statistically significant.

## Results

### Baseline characteristics

Of the 502 patients who received an ICD between 2015 and 2020 at the Radboudumc, 20 were excluded. Out of the remaining 482 patients, 257 (53.3%) patients underwent ICD implantation for primary prevention, and 225 (46.7%) patients had a secondary prevention indication. The baseline characteristics for all patients, also divided into primary or secondary prevention, are shown in Tab. 1. The median age was 66 (IQR 56–72) years. Ischaemic heart disease was most common in the secondary prevention group, while the LVEF was lower in the primary prevention group, with more patients suffering from non-ischaemic heart disease and heart failure symptoms. Furthermore, the two groups were significantly different regarding age, gender and body mass index. The primary prevention group also showed a higher prevalence of atrial fibrillation (30.4% vs 20.0%,* p* = 0.009), chronic renal disease (14.4% vs 5.8%, *p* = 0.002) and diabetes (27.6% vs 15.6%, *p* = 0.001). Patients in the secondary prevention group were significantly more likely to have a dual-chamber device (75.6% vs 38.5%, *p* < 0.001), whereas cardiac resynchronisation therapy was more prevalent in patients receiving an ICD for primary prevention (46.7% vs 7.1%, *p* < 0.001.

**Table 1:** Baseline characteristics for primary versus secondary prevention

Baseline characteristics	All patients (*n* = 482)	Primary prevention (*n* = 257)	Secondary prevention (*n* = 225)	*p*-value
*Demographics*				
Age (years), median (IQR)	66.0 (IQR 56.0–72.0)	67.0 (IQR 58.0–72.0)	65.0 (IQR 53.0–71.0)	0.043
Gender, male	377 (78.2%)	188 (73.2%)	189 (84.0%)	0.004
BMI (kg/m^2^), median (IQR)	26.9 (IQR 24.1–30.5)	27.4 (IQR 24.4–31.3)	26.4 (IQR 23.9–29.5)	0.016
*NYHA functional class*^*a*^				
I	208 (43.2%)	38 (14.8%)	170 (75.6%)	< 0.001
II	216 (44.8%)	174 (67.7%)	42 (18.7%)	< 0.001
III	56 (11.6%)	44 (17.1%)	12 (5.3%)	< 0.001
IV	1 (0.2%)	0	1 (0.4%)	0.468
*Medical history*				
Atrial fibrillation	123 (25.5%)	78 (30.4%)	45 (20.0%)	0.009
Cancer	63 (13.1%)	33 (12.8%)	30 (13.3%)	0.873
Cerebrovascular disease	71 (14.7%)	41 (16.0%)	30 (13.3%)	0.418
Chronic renal disease	50 (10.4%)	37 (14.4%)	13 (5.8%)	0.002
COPD	50 (10.4%)	29 (11.3%)	21 (9.3%)	0.483
Diabetes	106 (22.0%)	71 (27.6%)	35 (15.6%)	0.001
Peripheral vascular disease	33 (6.8%)	16 (6.2%)	17 (7.6%)	0.564
Heart valve replacement	39 (8.1%)	21 (8.2%)	18 (8.0%)	0.945
*History of cardiovascular disease*	453 (94.0%)	257 (100%)	196 (87.1%)	< 0.001
*Ischaemic heart disease*	266 (55.2%)	123 (47.9%)	143 (63.6%)	< 0.001
– Myocardial infarction	230 (47.7%)	106 (41.2%)	124 (55.1%)	0.899
– Coronary artery disease	36 (7.5%)	17 (6.6%)	19 (8.4%)	
– Coronary revascularisation	207 (42.9%)	99 (38.5%)	108 (48.0%)	0.331
*Non-ischaemic heart disease*	187 (38.8%)	134 (52.1%)	53 (23.6%)	< 0.001
– DCM	116 (24.1%)	101 (39.3%)	15 (6.7%)	< 0.001
– HCM	25 (5.2%)	18 (7.0%)	7 (3.1%)	0.967
– Electrical heart disease	4 (0.8%)	0	4 (1.8%)	0.006
– ARVD	8 (1.7%)	2 (0.8%)	6 (2.7%)	0.007
– Sarcoidosis	4 (0.8%)	3 (1.2%)	1 (0.4%)	1.000
– NCCM	1 (0.2%)	1 (0.4%)	0	1.000
– Non-specified non-ischaemic heart disease	29 (6.0%)	9 (3.5%)	20 (8.9%)	< 0.001
*Laboratory findings*				
eGFR (ml/min), median (IQR)	70.0 (IQR 57.0–87.5)	64.0 (IQR 52.3–79.0)	79.0 (IQR 63.0–90.0)	< 0.001
*Echocardiography*				
LVEF, median (IQR)	34.0 (IQR 27.0–49.3)	30.0 (IQR 25.0–33.0)	47.5 (IQR 35.0–55.0)	< 0.001
*Type of device*				
Single-chamber ICD (%)	61 (12.7%)	31 (12.1%)	30 (13.3%)	0.675
Dual-chamber ICD (%)	269 (55.8%)	99 (38.5%)	170 (75.6%)	< 0.001
CRT‑D (%)	136 (28.2%)	120 (46.7%)	16 (7.1%)	< 0.001
S‑ICD	16 (3.3%)	7 (2.7%)	9 (4.0%)	0.435
Follow-up, median (IQR)	2.4 (IQR 0.2–3.9)	1.9 (IQR 0.2–3.3)	2.7 (IQR 0.7–4.4)	< 0.001

Continuous variables are presented as mean ± SD or median (IQR) for skewed distributions, and categorical variables are presented as numbers (%)

*IQR* interquartile range, *BMI* body mass index, *NYHA* New York Heart Association, *COPD* chronic obstructive pulmonary disease, *DCM* dilated cardiomyopathy, *HCM* hypertrophic cardiomyopathy, *ARVD* arrhythmogenic right ventricular dysplasia, *NCCM* non-compaction cardiomyopathy, *eGFR* estimated glomerular filtration rate, *LVEF* left ventricular ejection fraction, *ICD* implantable cardioverter-defibrillator, *CRT‑D* cardiac resynchronisation therapy defibrillator, *S‑ICD* subcutaneous ICD

^a^One patient’s NYHA classification was missing

### Appropriate ICD therapy

During a median follow-up of 2.4 years (IQR 0.2–3.9), 62 secondary prevention patients (27.6%) received appropriate ICD therapy (ATP, ICD shock, or both) as first therapy compared to 23 (8.9%) in the primary prevention group (*p* = 0.003). When appropriate first ICD therapy was analysed for ATP or ICD shock separately, this difference remained. Comparison of ICD therapy for underlying cardiac pathology, ischaemic or non-ischaemic heart disease accordingly showed no differences. In all, 70.6% of the appropriate first therapy was administered for VT compared to 28.2% for VF.

### Time to appropriate ICD therapy

**Fig. 2:**
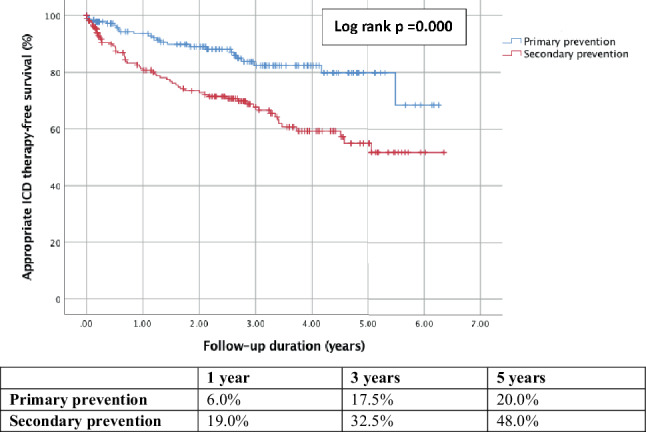
Kaplan-Meier curve of appropriate ICD therapy for primary versus secondary prevention and the cumulative incidences at 1, 3, and 5 years after device implantation

The Kaplan-Meier curve (Fig. 2) shows the time to appropriate ICD therapy since implantation compared for primary versus secondary prevention. This demonstrated that the cumulative incidence of appropriate therapy was 6.0% versus 19.0% for primary versus secondary prevention 1 year after the implantation. After 5 years, this cumulative incidence increased to 20.0% and 48.0% in the primary and secondary prevention group, respectively (*p* < 0.001).

### Predictors of appropriate ICD therapy

Possible predictors of appropriate ICD therapy during follow-up were analysed using binary logistic regression (Tab. 2). Univariable logistic regression showed several parameters being potential significant predictors of appropriate therapy during follow-up. In the binary, multivariable regression, only male gender [3.53, 95% confidence interval (CI) (1.003, 12.403), *p* = 0.049] and a secondary prevention indication [4.90, 95% CI (1.495, 16.066), *p* = 0.009] remained significant.

**Table 2:** Predictors of appropriate implantable cardioverter-defibrillator (*ICD*) therapy

	Univariable binary logistic regression		Multivariable binary logistic regression, enter method		Multivariable binary logistic regression, forward LR	
	OR	(95% CI)	Exp(B)	(95% CI)	OR	(95% CI)
Constant			*0.26*		*0.037*	
Gender	1.94	(1.009, 3.720)*	3.53	(1.003, 12.403)*	3.50	(1.118, 10.974)*
eGFR	1.02	(1.007, 1.036)**	1.02	(0.987, 1.056)		
LVEF	1.02	(1.002, 1.036)*	1.01	(0.960, 1.057)		
Indication	3.50	(2.110, 5.803)**	4.90	(1.495,16.066)**	6.22	(2.665,14.502)**
BMI	0.92	(0.873, 0.971)**	0.93	(0.843, 1.026)		
DCM	0.26	(0.114, 0.577)**	0.60	(0.170, 2.149)		
ARVD	5.56	(1.309, 23.571)*	2.46	(0.450, 13.463)		
NYHA I	2.00	(1.250, 3.204)**	0.25	(0.046, 1.327)		
NYHA II	0.41	(0.243, 0.675)**	0.31	(0.072, 1.309)		
Dual-chamber ICD	1.73	(1.059, 2.810)*	0.49	(0.137, 1.786)		
CRT‑D	0.48	(0.264, 0.871)*	0.72	(0.162, 3.191)		
Nagelkerke R squared			0.310		0.232	

*OR* odds ratio, *CI* confidence interval, *LR* likelihood ratio, *eGFR* estimated glomerular filtration rate, *LVEF* left ventricular ejection fraction, *BMI* Body Mass Index, *DCM* dilated cardiomyopathy, *ARVD* arrhythmogenic right ventricular dysplasia, *NYHA* New York Heart Association, *CRT*-*D* cardiac resynchronisation therapy defibrillator

*Significant (0.01 < *p* < 0.05)

**Significant (*p* < 0.01)

### Inappropriate ICD therapy

Analysis of the first ICD therapy showed that 13 (5.1%) patients with a primary prevention indication had inappropriate ICD therapy as first therapy compared to eight (3.6%) secondary prevention patients (*p* = 0.003). However, rates of inappropriate ICD shock were low in both groups. Six patients (2.3%) versus one patient (0.4%) received inappropriate first ICD shock in the primary versus secondary prevention group, respectively (*p* = 0.174) (Tab. 3). Inappropriate first ICD therapy (ATP, ICD shock, or both) occurred 21 times in total; in 18 cases this was due to a non-ventricular arrhythmia. In the remaining cases, inappropriate therapy was administered because of a device-related- or technical problems.

**Table 3:** Study outcomes for primary versus secondary prevention

Study outcomes	All patients (*n* = 482)	Primary prevention (*n* = 257)	Secondary prevention (*n* = 225)	*p*-value
*Incidence of first ICD therapy (%)*				
Appropriate first ICD therapy (shock and ATP)	85 (17.6%)^a^	23 (8.9%)	62 (27.6%)	0.003
Appropriate first ICD shock	44 (9.1%)^b^	14 (5.4%)	30 (13.3)	0.011
Appropriate first ATP	49 (10.2%)^b^	11 (4.3%)	38 (16.9%)	0.026
Inappropriate first ICD shock	7 (1.5%)	6 (2.3%)	1 (0.4%)	0.174
*Complications* (%)	81 (16.8%)	42 (16.3%)	39 (17.3%)	0.772
*Mortality and hospitalisation* (%)				
Hospitalisation for cardiovascular reason	154 (32.0%)	75 (29.2%)	79 (35.1%)	0.559
All-cause mortality	58 (12.0%)	32 (12.5%)	26 (11.6%)	0.763

Categorical data are presented as numbers (%)

*ICD* implantable cardioverter-defibrillator, *ATP* anti-tachycardia pacing

^a^ No. of patients with appropriate first ICD therapy = 85

^b^ No. of patients with appropriate first ICD shock + first ATP (44 + 49) = 93. This difference is because eight patients received ATP with an ICD shock as appropriate first therapy, which was counted as 1

### Complications

The overall occurrence of complications did not differ significantly between the primary and secondary prevention indications (16.3% vs 17.3%, *p* = 0.772). Access-related and lead-related complications were the most common. Access-related complications were reported in 14 (5.4%) of the primary prevention patients compared to 18 (8.0%) in the secondary prevention group (*p* = 0.238). The number of lead-related complications in primary versus secondary prevention patients was 22 (8.6%) and 16 (7.1%), respectively (*p* = 0.306).

### Hospitalisation and all-cause mortality

**Fig. 3:**
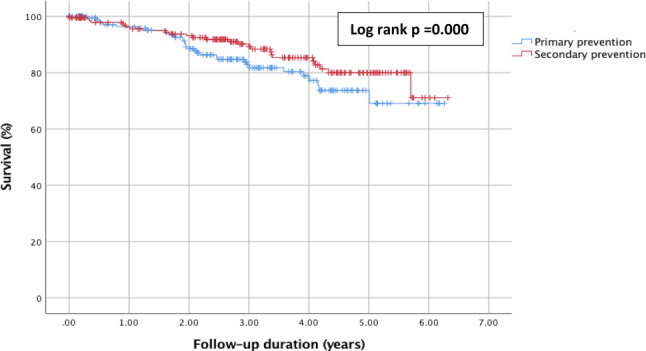
Kaplan-Meier curve of all-cause mortality for primary versus secondary prevention

Hospitalisation for a cardiovascular reason during follow-up was similar for the two groups, with 75 (29.2%) cases in the primary prevention group and 79 (35.1%) in the secondary prevention group (*p* = 0.559). Regarding all-cause mortality, 58 (12.0%) patients died in total, with 32 (12.5%) and 26 (11.6%) patients in the primary and secondary prevention group, respectively (*p* = 0.763) (Tab. 3). Among these rates, death before first therapy occurred in 39 patients with no difference between the prevention indications (*p* = 0.283). Three out of the ten cardiac deaths in the secondary prevention group, compared to one out of the ten cardiac deaths in the primary prevention patients, were arrhythmic deaths. Sixteen cardiac deaths were due to end-stage heart failure, stent thrombosis, or a ruptured aneurysm. In six out of these 16 patients, the device was deactivated. Figure 3 shows the Kaplan-Meier survival curve for the time to mortality (cardiac and non-cardiac) since device implantation. The survival curves were similar when analysed according to the indications (*p* = 0.157).

## Discussion

The results of this study indicated that a higher number of secondary prevention patients received appropriate (first) ICD therapy. These patients also received appropriate therapy within a significantly shorter time period after device implantation. This difference remained when the appropriateness of the first ICD therapy was analysed for ATP or ICD shock separately. Whereas inappropriate first therapy was more often seen in patients with an ICD for primary prevention, the rates of inappropriate shock as first therapy were comparable and low. These findings are in line with results from previous studies. Konstantino et al. analysed ICD therapy for primary versus secondary prevention. Their comparison showed that patients with a secondary prevention indication had a higher chance of receiving appropriate ICD therapy (27% vs 18%, during seven years of follow-up) with a shorter time from implantation to first appropriate therapy (*p* = 0.021) [3]. Bae et al. also concluded that appropriate therapy was more often seen in secondary prevention patients (34.8% vs 18.0%, *p* = 0.001) [6].

This current study showed no differences in the appropriate first ICD therapy for different underlying cardiac pathologies such as ischaemic or non-ischaemic heart disease. Bae et al. analysed ICD therapy according to the indication and aetiology. Their baseline characteristics also showed a lower prevalence of ischaemic heart disease among primary prevention patients, with more patients in the higher NYHA functional classes (2.5 ± 0.8 vs 1.9 ± 0.8, *p* < 0.001). Furthermore, the results showed no differences in ICD therapy between ischaemic and non-ischaemic aetiology [6]. This is in line with the results of our study.

The all-cause mortality rates, including cardiac and non-cardiac death, did not differ between patients with a primary or secondary prevention indication. These data aere supported by several previous studies [3, 5, 6] Multivariable binary logistic regression showed that male gender and a secondary prevention indication were predictors of appropriate ICD therapy, also when correcting for follow-up duration. The likelihood of receiving appropriate therapy was 3.53 times larger for the male gender than for the female gender (*p* = 0.049) and 4.9 times larger for secondary prevention compared to primary prevention (*p* = 0.009), predictors that have previously been found to be comparable [3, 4].

In 70% of cases, appropriate ICD therapy was given for VT; hence therapeutic options such as medication, ablation and even surgery should be considered and could prevent a large proportion of ICD therapy in the future, most importantly shocks. Substrate ablation, in particular, has been evaluated for the last decade and has shown good VT-free survival at mid-term follow-up [7–12].

### Limitations

The retrospective study design is a limitation of this study. Since the Radboudumc is a referral hospital, a proportion of the patients were seen at outpatient clinics of the referring hospitals. Remote monitoring of these patients was done at the Radboudumc. The influence of different ICD programming strategies and the availability of different treatment options (medical and interventional) over time should be taken into consideration, as these limit the results of this retrospective study to a certain extent. Another limitation of a retrospective analysis is the possibility of missing data.

## Conclusion

The risk associated with appropriate (first) ICD therapy is higher in patients with an ICD for secondary prevention, and these patients receive this treatment within a shorter time frame after device implantation compared to patients with a primary prevention indication. The occurrence of complications, hospitalisation for cardiovascular disease and all-cause mortality does not differ significantly between the two groups.
